# High *ex vivo* recombination rate of African swine fever virus in porcine alveolar macrophages

**DOI:** 10.1080/01652176.2026.2740247

**Published:** 2026-10-23

**Authors:** István Mészáros, Ferenc Olasz, István Miklós, Eszter Göltl, Barbara Oláh, Zoltán Zádori

**Affiliations:** a HUN-REN Veterinary Medical Research Institute, Budapest, Hungary; b HUN-REN Alfréd Rényi Institute of Mathematics, Budapest, Hungary; c HUN-REN Institute for Computer Science and Control, Budapest, Hungary

**Keywords:** Co-infection, homologous recombination, recombination frequency, African swine fever virus, vaccine safety, DIVA, porcine alveolar macrophage (PAM), viral recombination

## Abstract

Using fluorescently labeled African Swine Fever Virus (ASFV) strains, a high (~0.2) quasi-recombinant frequency (qRF^3-4^) was detected *ex vivo* during 3–4 replication cycles in naïve porcine alveolar macrophages (PAM). While co-infection and recombination frequencies remained low in PAM, they increased sharply between the second and fourth viral cycles. Infection multiplicity and synchronicity significantly influenced ASFV recombination frequency, which correlated positively with genetic distance. Crossing the ASFV/Lv17/WB/Rie1 mutants yielded a saturation curve resembling the Haldane mapping function, plateauing at ~20% recombination frequency at 160 kbp. Crossing less homologous strains (ASFV/Lv17/WB/Rie1 × ASFV/NHV/P68) reduced the recombination frequency; however, at large marker distances (140 kbp), it approached values (0.18) observed in homologous strains. A novel mathematical model estimated the average co-infection rate to be 0.47 after three *viral* cycles. Empirical application of the model revealed that 1 centimorgan translates to ~550–1650 bp, meaning offspring virions from a single cell undergo an average of 1.15–3.45 recombination events. This high recombination rate is consistent with *in vivo* observations and has critical implications for ASFV prevention and vaccination strategies. Such genomic plasticity can facilitate rapid viral evolution, potentially undermining the efficacy of live-attenuated vaccines globally.

## Introduction

Since its emergence in Georgia in 2007, African Swine Fever Virus (ASFV) has spread extensively across Eurasia, severely impacting the global swine industry (Rowlands et al. 2008). Despite over 50 years of research, the development of a safe and effective vaccine remains challenging due to the virus' complexity (Alejo et al. 2018). Traditional inactivated vaccines have proven ineffective, and modern subunit or recombinant methods targeting various ASFV antigens have only provided insufficient protection against disease (Dong et al. 2026). The immunogenic structural proteins (e.g. p54, p30, and p72) expressed in different systems were either only partially protective or ineffective in challenge experiments (Neilan et al. 2004). Recombinant adenovirus and vaccinia virus vectors expressing different ASFV antigens (up to 35 ASFV proteins) in various combinations successfully elicited both cellular and humoral immune responses, but they provided only marginal protection in animals (Lacasta et al. 2014; Lokhandwala et al. 2016; Arias et al. 2017; Cadenas-Fernández et al. 2020; Zajac et al. 2023). Since live attenuated vaccines appear to be the most effective tool for controlling the ASFV pandemic, current vaccine research primarily focuses on utilizing genome editing to knock out non-essential genes (Afonso et al. 1998; O’Donnell et al. 2015; Sanford et al. 2016; O’Donnell et al. 2017; Borca et al. 2020; Franzoni et al. 2023; Tamás et al. 2023; Petrini et al. 2023a) that influence virulence and the immune response, thereby creating effective modified live virus (MLV) vaccines.

A primary safety concern regarding MLVs is the inherent risk of genetic recombination with circulating field isolates or other vaccine strains. Such events may facilitate the emergence of novel serotypes, genotypes, or pathotypes, potentially leading to vaccine-associated adverse events or outbreaks (Fan et al. 2024). Consequently, a comprehensive understanding of the mechanisms underlying these recombination events is critical for risk mitigation and the effective deployment of ASFV MLV vaccines.

Even though homologous recombination is known to play an important role in the evolution of large DNA viruses such as herpes and poxviruses (Gershon et al. 1989; Umene 1999; Qin and Evans 2014), little is known about this process in ASFV. Indirect experimental evidence, such as the successful use of the CRISPR/Cas system to produce recombinant viruses, and *in silico* data, suggest that homologous recombination may contribute to the genetic diversity of ASFV. Recombination events have repeatedly been detected in specific loci, including the *MGF360*, *E183L*, *B602L*, *B646L*, *CP204L*, *EP153R,* and *EP402R* (*CD2v*) genes (Chapman et al. 2008; Michaud et al. 2013; Nefedeva et al. 2020). Furthermore, a comprehensive bioinformatics analysis of 39 complete ASFV genomes (Zhu et al. 2019) revealed multiple potential recombination events in each of them.

In the last 3 years, we (Petrini et al. 2023a) and others (Ramirez-Medina et al. 2024; Kitamura et al. 2025; Ma et al. 2025; Tran et al. 2025; Li et al. 2026) successfully isolated recombinant ASFVs from both porcine alveolar macrophages (PAMs) cultured *ex vivo* and pigs, providing direct experimental evidence for homologous recombination between ASFV strains.

In the present study, we used several fluorescently labeled, genetically engineered ASFV strains to characterize their *ex vivo* homologous recombination across 3‒4 viral cycles in PAMs, defining the corresponding quasi-recombination frequencies. Our findings reveal novel aspects of ASFV recombination and provide robust empirical evidence of the high recombinogenic potential of ASFV during intracellular replication.

## Materials and methods

### Cells and viruses

Viruses were cultured in PAMs. The cells were isolated from pig lungs following the protocol of the WOAH Manual (World Organisation for Animal Health, 2019) and were stored at −80 °C in RPMI-1640 medium (Thermo Fisher Scientific, Waltham, MA, USA) with 30% bovine serum albumin (Capricorn Scientific, Ebsdorfergrund, Germany) and 10% DMSO. PAMs were cultured in RPMI-1640 medium with 10% bovine serum albumin, 1% sodium-pyruvate (Thermo Fisher Scientific, Waltham, MA, USA), 1% antibiotic solution (Thermo Fisher Scientific, Waltham, MA, USA) and 1% non-essential amino acids (Thermo Fisher Scientific, Waltham, MA, USA) (Olasz et al. 2020) at 37 °C in 5% CO_2_.

The biotype II strains ASFV/HU/2018 (MN715134.1) (Olasz et al. 2019) and ASFV/Lv17/WB/Rie1 (OR863253.1) (Gallardo et al. 2019) and the biotype I strain ASFV/NHV/P68 (KM262845.1) were used to generate knock-out strains (Dinhobl et al. 2023). Next to the novel knock-out strains ASFV/HU/2018/ΔCD (PZ387669) and ASFV/NHV/P68/ΔI177L (PZ387670), also the previously published mutant strains ASFV/Lv17/Δ110-11L (OR872427.1) (Tamás et al. 2023), ASFV/Lv17/ΔGL (OR872426.1) (Petrini et al. 2023a), ASFV/Lv17/ΔCD (PQ284530.1), ASFV/Lv17/ΔEP (PQ284532.1), ASFV/Lv17/ΔUK (PQ284533.1) and ASFV/Lv17/ΔCD/ΔUK (PQ284531.1) (Gallardo et al. 2024) were used as parental strains in recombination experiments (Table 1). The abbreviations Lv17, Hu, and NHV are used for the ASFV/Lv17/WB/Rie1, ASFV/HU/2018, and ASFV/NHV/P68 strains, respectively, throughout the paper.

**Table 1.** List of the parental ASFV strains.

Parental strain	Strain referenced as	Deleted gene	Inserted marker gene	GenBank accession number
ASFV/HU/2018/ΔCD	Hu/ΔCD	*EP153R, EP402R*	eGFP	PZ387669
ASFV/Lv17/WB/Rie1/ΔCD	Lv17/ΔCD	*EP153R, EP402R*	eGFP	PQ284530.1
ASFV/Lv17/WB/Rie1/ΔEP	Lv17/ΔEP	*EP153R*	eGFP	PQ284532.1
ASFV/Lv17/WB/Rie1/ΔGL	Lv17/ΔGL	*B119L*	mCherry	OR872426.1
ASFV/Lv17/WB/Rie1/ΔUK	Lv17/ΔUK	*DP96R*	DsRed	PQ284533.1
ASFV/Lv17/WB/Rie1/Δ110-11L	Lv17/ΔG	*MGF 110-11L*	eGFP	OR872427.1
ASFV/Lv17/WB/Rie1/ΔCD/ΔUK	Lv17/ΔCD/ΔUK	*EP153R, EP402R, DP96R*	eGFP, DsRed	PQ284531.1
ASFV/NH/P68/ΔI177L	NHV/ΔI177L	*I177L*	mCherry	PZ387670

### Construction of recombinant ASFV strains

Single gene knock-out (sgKO) viruses were generated by CRISPR/Cas9-mediated homologous recombination, similarly as previously described (Borca et al. 2018; Tamás et al. 2023).

Transfer plasmids were prepared from transfer cassettes and the pUC19 vector (Thermo Fisher Scientific, Waltham MA, USA). Transfer cassettes were constructed from the left homologous arm, the ASFV p72 promoter, the marker gene (eGFP, DsRed or mCherry) and the right homologous arm. To create the transfer plasmid, the linearized pUC19 plasmid and the three overlapping PCR fragments of the transfer cassette were assembled using the Seamless PLUS Cloning and Assembly Kit (Thermo Fisher Scientific, Waltham MA, USA). The two gRNA plasmids were generated by cloning double-stranded protospacer oligonucleotides targeting upstream and downstream regions into the BbsI-digested pX330-DNLS1_2-NeoR plasmid (Barasona et al. 2021). A total of 3 × 10^5^ PAMs were seeded in a 6-well plate and incubated for 24 h at 37 °C and 5% CO_2_. Next, 1.5  μg transfer plasmid and 0.5  μg per each of gRNA plasmids were mixed with 10  μl FuGENE® HD transfection reagent (Promega, Madison, USA) and 150  μl RPMI-1640. The transfection reagent/DNA complex was incubated for 10 min. at room temperature before being added to PAMs. Immediately following transfection, the cells were infected with the parental ASFV strain at a multiplicity of infection (MOI) of 1 and incubated for 24 h at 37 °C and 5% CO_2_.

After incubation, the culture medium was removed from the wells, and PAMs expressing the marker protein were sorted using an automated pipette under an Axio Observer D1 inverted fluorescence microscope (Carl Zeiss Ag. Oberkochen, Germany). The isolated cells underwent three freeze‒thaw cycles, were adjusted to a final volume of 100 μl with culture medium, and were subsequently used to infect freshly seeded PAMs. The ratio of the parental-mutant ASFV-infected cells was detected at each infection step using immunofluorescence staining (Szeredi et al. 2020). Once only the mutant virus was detected, sample purity was confirmed by sequencing following a previously published protocol (Olasz et al. 2019).

### Co-infection experiments

Freshly seeded PAMs were incubated for 24 h at 37 °C and co-infected with two parental strains (both at an MOI of 3), each carrying a different fluorescent marker gene. The medium was changed after 1 h of incubation (Figure 1).

The number of parental virus-infected cells (green or red only) and co-infected/recombinant virus-infected cells (red-green or colorless) was monitored using immunofluorescence (IF) staining over a period of 72 h. Infected cells were detected using mouse anti-p72 antibodies and Cy5™ goat anti-mouse IgG (H+L) (Thermo Fisher Scientific, Waltham, MA, USA), while nuclei were stained using Hoechst reagent. Each co-infection was repeated at least three times. The cells were monitored and photographed using an Axio Observer D1 inverted fluorescence microscope (Carl Zeiss AG, Oberkochen, Germany). Red and green fluorescent signals were detected using Zeiss filter sets 31 Alexa Fluor 568 and 38 HE GFP, respectively. To exclude false-positive signal detection, exposure times of 110–220 ms were used for the green channel, while exposure times of 100–400 ms were applied for the red channel. The cells were counted using the ImageJ program (Grishagin 2015).

**Table 2.** List of ASFV parental strains used in the cross experiments and the distance (nt) between the deleted genes.

strain I	strain II	deleted genes	distance (nt)
Lv17	Lv17	dCD/dGL	20500
Lv17	Lv17	dEP/dGL	21652
Lv17	Lv17	dG/dGL	75839
Lv17	Lv17	dCD/dUK	109914
Lv17	Lv17	dEP/dUK	111066
Lv17	Lv17	dG/dUK	165253
Lv17	NHV	dCD/I117L	85038
Lv17	NHV	dEP/I177L	86190
Lv17	NHV	dG/I177L	140377
Lv17	Hu	dCD-dGL/wt	21652
Lv17	Hu	dCD-dUK/wt	111066
Lv17	Lv17	dCD-dUK/wt	109914

### Quasi recombination frequency

Freshly seeded PAMs (10^5^ cells) in a 24-well plate were incubated for 24 h at 37 °C. Then, they were co-infected with two parental strains carrying different fluorescent marker genes (Table 2) at a multiplicity of infection (MOI) of 3 for both strains. The medium was replaced with fresh medium after 1 h of incubation. The cells were incubated at 37 °C, and then the viruses were harvested at 3 days post-infection (dpi). The titer (fluorescent focus unit: FFU/ml) of the parental (red or green) and recombinant viruses (red-green or colorless) was determined from the supernatant by IF (see above).

The recombination frequency (RF) is used to quantify the physical distance and the genetic linkage between markers in a genome. The RF is usually calculated from the number of recombinant offspring produced within a single generation as follows: RF = number of recombinant offspring/total number of offspring.

Owing to the extreme difficulty of calculating the recombination frequency in one generation, and the uneven temporal distribution of the co-infection and recombination events in tissue culture, the decision was made to quantify the recombination frequency at 72 hpi after 3‒4 virus replication cycles. Since this unit of measurement differs from the classic RF measured within one generation, it was named quasi-recombination frequency (qRF^3-4^).

The qRF^3-4^ was calculated by dividing the titer of the recombinants by the total titer of viruses and multiplying by 100 at 72 h post-infection (hpi) after 3–4 virus replication cycles (Figure 1). To reduce the possibility of misidentifying a large number of co-infected cells as infected with recombinant virus, we performed tenfold serial dilutions of the parental mix, plated them on PAMs, and fixed them at 20 hpi.

**Figure 1.**
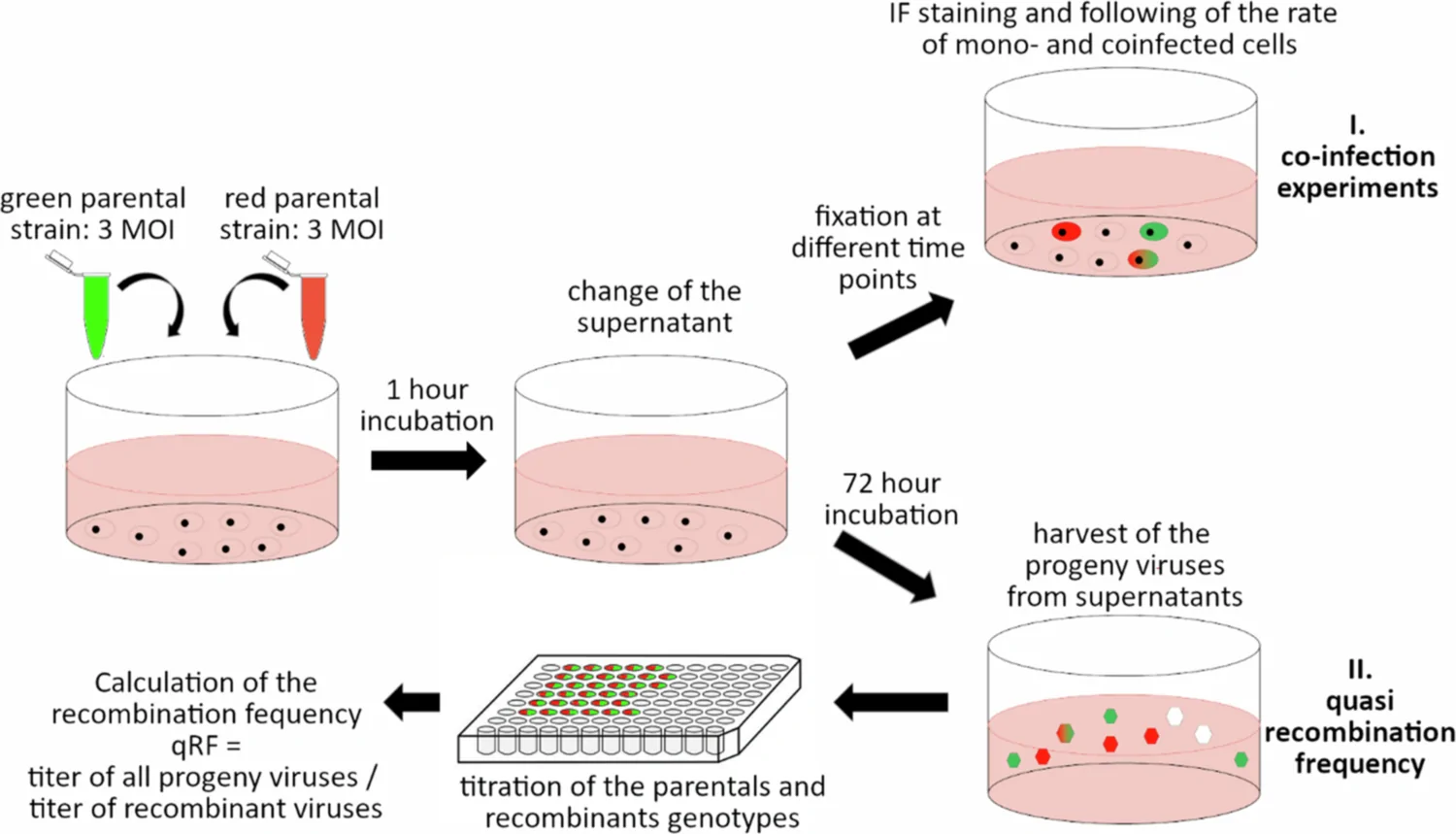
Flowchart of the determination of co-infection and the quasi recombination frequency (qRF^3-4^). The red–green cells (I) represent co-infection, while the red–green and white viruses (II) represent recombinant progeny viruses.

### Infection time and multiplicity dependence of recombination frequency

For investigating the time dependence of co-infection, PAMs were infected at 24-hour post-plating with either the Lv17/ΔEP or Lv17/ΔGL strain (see Table 1) at an MOI of 3 for both strains. The supernatant was replaced after 1  h of incubation. At 2, 4, and 6 h after the initial infection, the cells were re-infected with the other parental virus. To maintain identical conditions, the supernatants were replaced 1 h after each infection. The supernatants were harvested 72 h after the first viral infection. Detection of recombinants and calculation of RF^3-4^ were performed as described above.

To examine the dependence of qRF^3-4^ on the MOI, PAMs were co-infected with the Lv17/ΔCD and Lv17/ΔUK strains at MOIs of 0.3–0.3, 1–1, and 3–3. The supernatant was replaced after 1 h of incubation. The qRF^3-4^ value was determined after 72 h by titration, as described above.

### Statistical analysis and calculation of map distance

The normality of the distribution of residuals was determined using the Shapiro–Wilk test. The RF of the different crosses was compared using a two-sample t-test. Online calculators were used for the statistical analysis (https://www.statskingdom.com/index.html). The calculation of map distance was based on Haldane’s mapping function.

### Modeling PAMs co-infection

The simulation and visualization were implemented in Python (Python Software Foundation 2016). The main entry point of the script is the plot_results() function, which calls the simulation routine (simulate()) to compute recombination frequencies across a range of genetic distances (1–300 cm). The function then generates a publication-quality plot using the matplotlib library, displaying total recombination as well as the contributions of the recombinant (red-green) and wild-type genotypes.

The plot is formatted with standardized axis labels, limits, and styling suitable for publication. Upon execution, the script saves the resulting figure both as a vector graphic (figure_recombination.pdf) and as a high-resolution raster image (figure_recombination.png, 300 dpi), and displays it interactively. The program can be run as a standalone Python script and requires only standard scientific Python dependencies (matplotlib) (Hunter 2007).

## Results

### Co-infection in PAMs

A suitable option for studying ASFV recombination *ex vivo* is to cross viruses that carry fluorescent protein marker genes. Cells infected with such viruses are easily identifiable under a fluorescence microscope, and cells infected with recombinant viruses can be readily distinguished from the parental viruses. Six marker gene-modified Lv17 viruses were already at our disposal (Table 1), but to examine the effects of genetic distance and DNA homology, as well as to obtain a more detailed understanding of homologous recombination in ASFV, additional fluorescent marker mutants of a biotype I (NHV/ΔI177L) and a biotype II strain (HU/ΔCD) were created. A total of nine parental viruses were used for the recombination experiments (Figure 2, Table 1).

**Figure 2.**
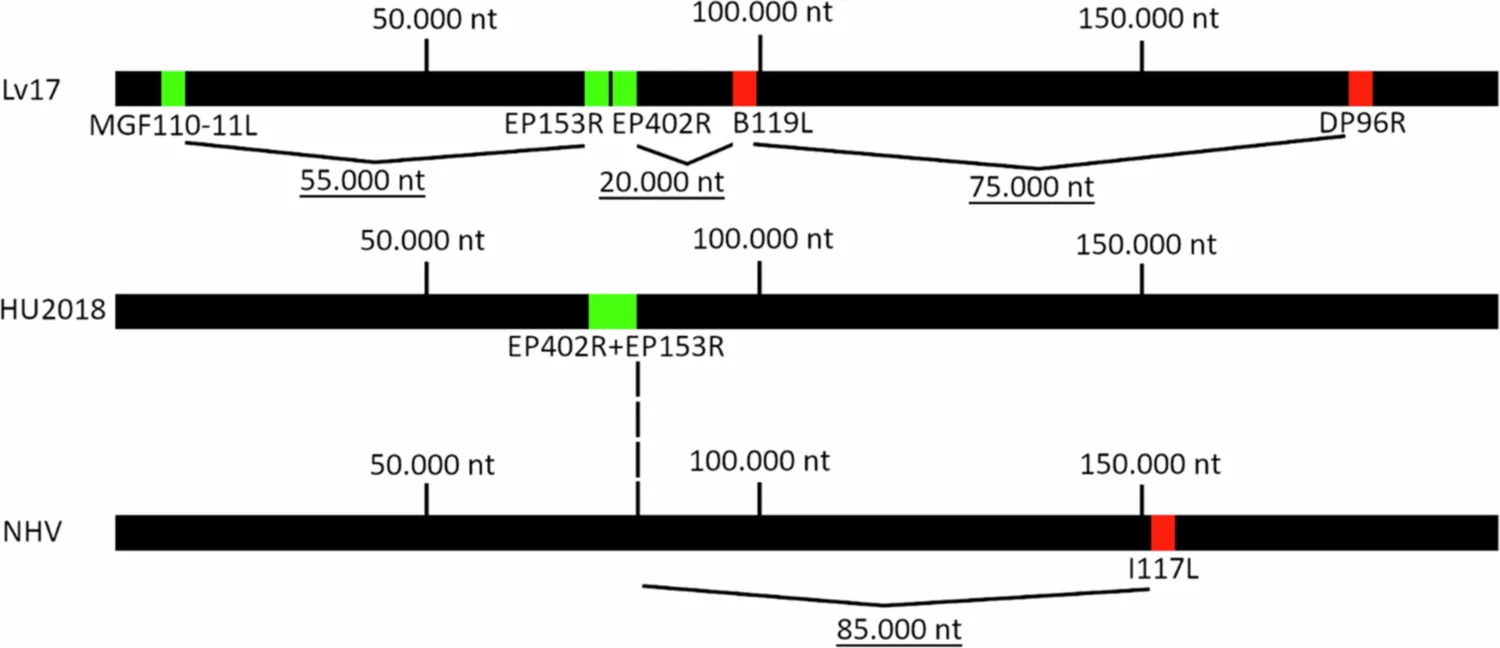
Location of the fluorescent marker genes in the genome of different ASFV strains. The green color represents the enhanced green fluorescent protein (eGFP_, and the red color marks the DsRed protein (in the case of the *DP96R* deletion) or the mCherry protein. The underlined numbers show the approximate distance (nt) between the markers. The Lv17 pictogram represents five different *single* knockout and one double knockout (Δ*EP402R*-Δ*DP96R*) strains.

Only a few established cell lines are reported to be sensitive to ASFV infection, but they are not available to us or the majority of ASFV researchers. Therefore, we chose PAMs for the recombination experiments as they are the primary target cells of ASFV *in vivo*. They can be infected by all biotypes, making them suitable for simulating *in vivo* cellular conditions during viral replication.

To study recombination, we performed co-infection experiments with two parental viruses and followed the infection process over time by observing fixed cells using a fluorescence microscope at regular intervals. In the first viral cycle, at a relatively high multiplicity of infection (MOI of 3 + 3), only 7.81%–11.86% of cells were positive for ASFV 16–24 h post-infection, and an even smaller proportion of naïve PAMs (~0.82%–0.93%) were co-infected (Figure 3A,C). In later cycles (at 48 hpi), there was a sharp increase in the rate of infected cells (32.78%–33.21%) (Figure 3A,C), as well as in the proportion of cells co-infected or infected with a recombinant virus (32.46%–41.21%) (Figure 3B,D). Notably, the recombinant green-red virus-infected cells were indistinguishable from those co-infected with the green and red parental viruses. By 72 hpi, virtually all cells had detached from the plate owing to infection-induced cell death and the bystander effect.

These data suggest that naïve cells are less susceptible to ASFV infection than are uninfected cells in an already infected culture, and that recombination and co-infection rates increase sharply during the second to fourth viral cycles.

**Figure 3.**
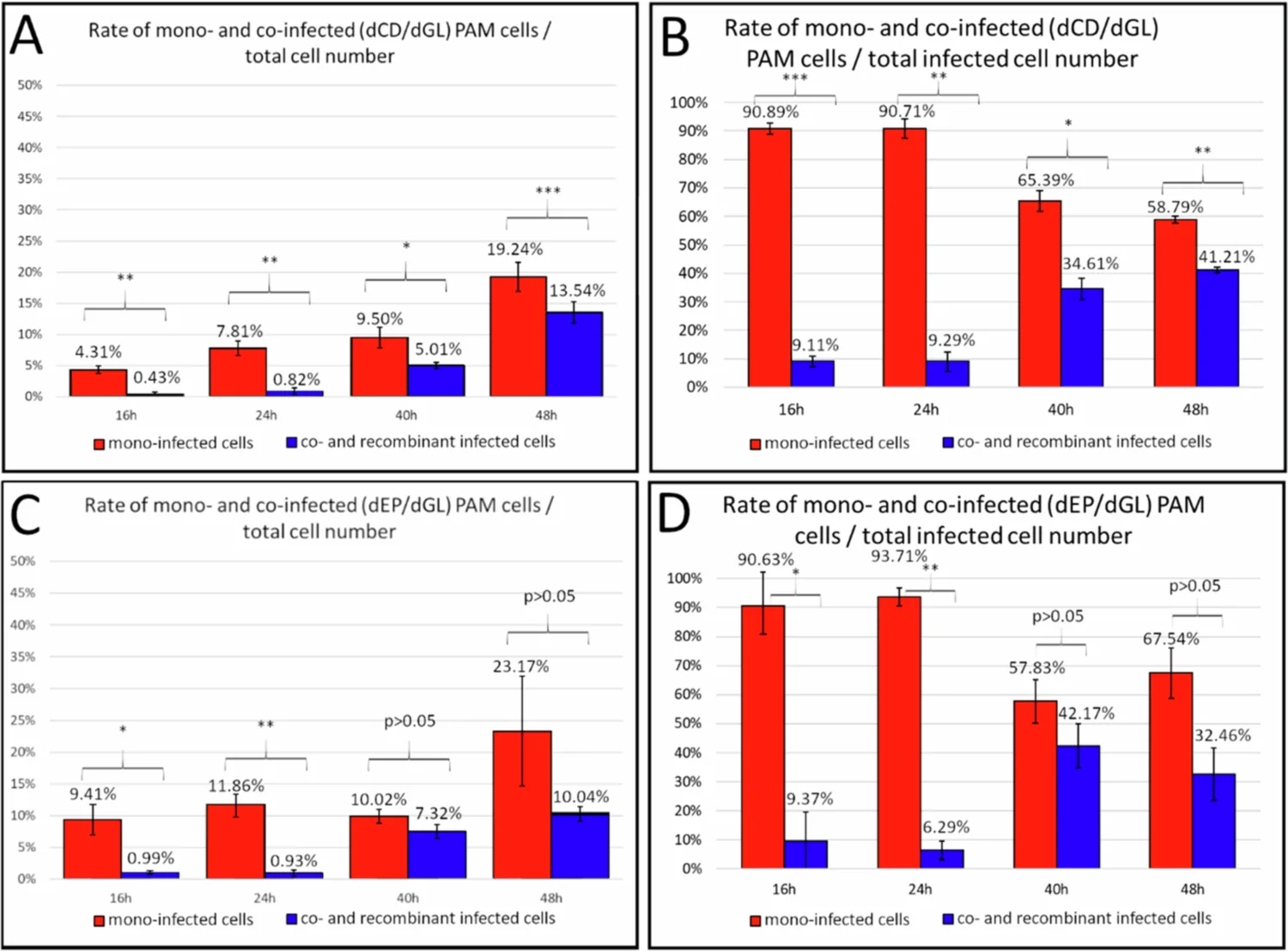
Changes in the rate of mono- and co-infected cells during an infection with two different parental strains. The red color represents infections with parental strains, while blue indicates co-infections or recombinant strains. Each measurement was performed in triplicate. Statistical significance was evaluated using paired-samples t-tests. *: *P* ≤ 0.05; **: *P* ≤ 0.01; ***: *P* ≤ 0.001. Lv17/ΔCD × Lv17/ΔGL cross: (A) The rate of infected cells as a function of total cell number and (B) as a function of total infected cell number. Lv17/ΔEP × Lv17/ΔGL cross: (C) The rate of infected cells as a function of total cell number and (D) as a function of total infected cell number.

### Factors influencing recombination frequency

Determining RF is generally difficult for eukaryotic viruses, and it is particularly challenging in the case of ASFV. One of the primary problems is that naïve PAMs cannot be 100% infected with ASFV within a single viral cycle (16–24 h). An additional serious challenge arising from this is that, in the case of high-multiplicity infections, the number of progeny viruses after one viral cycle does not significantly exceed the number of parental viruses remaining on the plate from the inoculum. Furthermore, it is impossible to distinguish progeny viruses that originated from co-infected cells (where recombination between the two parental strains can occur) from those that originated from cells infected with only one of the parental strains.

Because of the extreme difficulty of calculating the recombination frequency within one generation, and the uneven temporal distribution of the co-infection and recombination events in tissue culture, it was decided to quantify the recombination frequency at 72 hpi, representing 3–4 virus infection cycles. Since this unit of measurement differs from RF, which is calculated on the basis of one virus infection cycle, we named it quasi-recombination frequency (qRF^3-4^). For qRF^3-4^ determination, cell culture supernatants were collected at 72 hpi. Parental and recombinant viral loads were quantified by titration using serial dilutions, with cell fixation performed at 20 hpi to restrict analysis to a single round of replication. It could be argued that using this method, cells co-infected with viruses of both parental strains (red-green) might interfere with the accurate determination of the recombinant titer (also red-green). However, we observed that the proportion of co-infected cells in a naïve cell population remained low even in high-multiplicity infections up to 24  h (Figure 3). Furthermore, the serial dilution performed during titer determination reduces the number of co-infected cells, essentially eliminating this background. This was confirmed by the finding that, in a 1:1 mixture of parental strains (Lv17/ΔEP and Lv17/ΔUK) crossed at different MOIs (6–0.01), no co-infected cells were detected at 20 hpi at 0.1 MOI and below (Figure 4). Therefore, infected cells showing both red and green fluorescence detected at higher dilutions in virus titrations must originate from true recombinant viruses.

**Figure 4.**
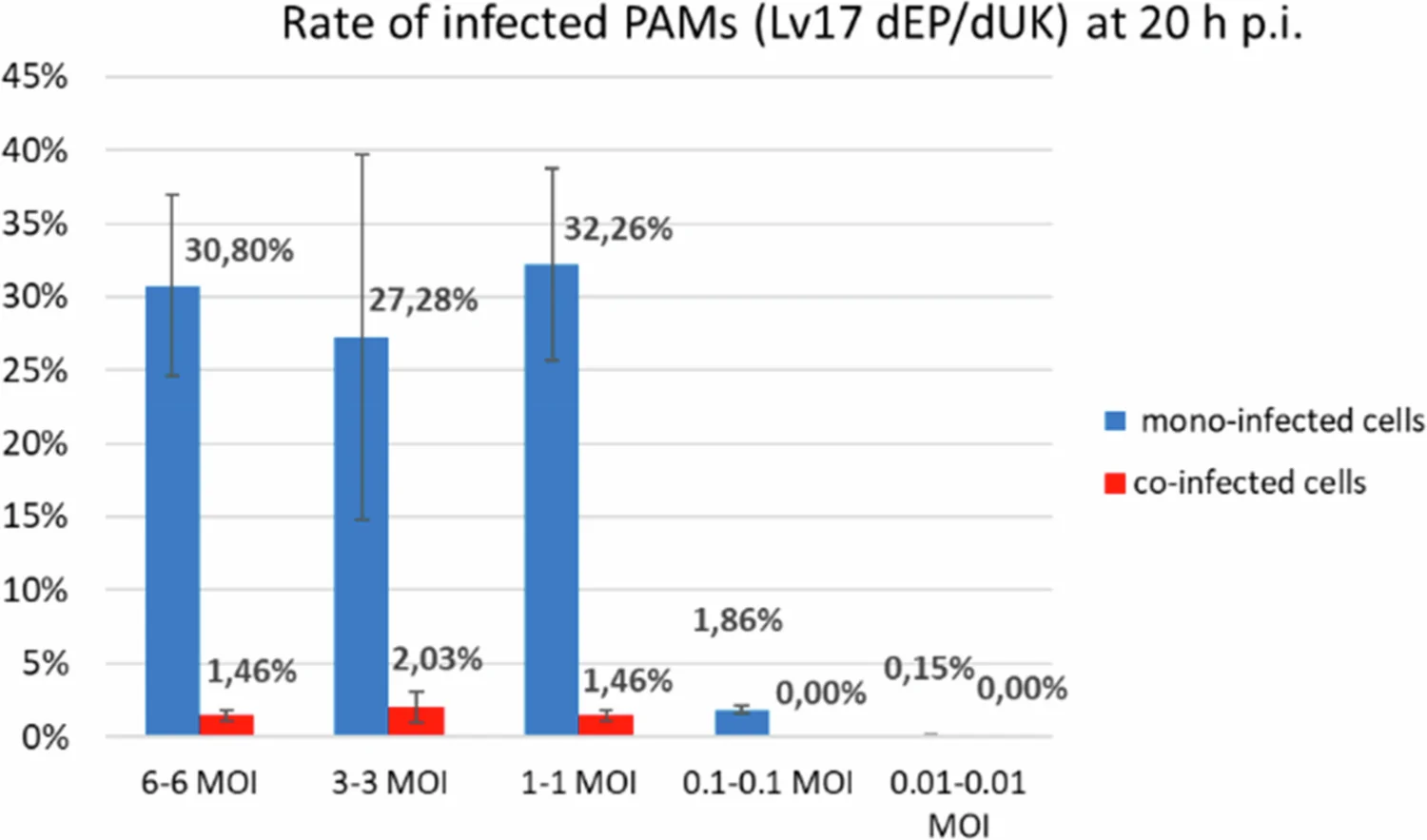
The rate of co-infected cells depends on the multiplicity of infection at 20 hpi. PAMs were infected with the Lv17/ΔEP and Lv17/ΔUK strains. The indicated rates are averages based on a minimum of three individual replications. (Note: the infection rate of naïve PAMs at 20 hpi can vary between ~10% and 35% from experiment to experiment).

Further investigation of the experimental conditions revealed that the MOI significantly influenced not only the results of the 20-hour co-infection experiment described above, but also those of 72-hour co-infection experiments. For example, in the Lv17/ΔEP × Lv17/ΔGL co-infection experiment, a 10-fold decrease in multiplicity resulted in a 6-fold decrease in the proportion of recombinants (Figure 5A). The timing of infections also appeared to be a major factor influencing the observed proportion of recombinants; a 2-hour deviation from synchrony caused a drastic, almost 10-fold decrease in the proportion of recombinants in the Lv17/ΔEP × Lv17/ΔGL co-infection experiment, regardless of which virus was first used for infection (Figure 5B).

To provide further evidence that the co-infection background is negligible in interpreting the results, we performed a ‘reciprocal cross’ of the Lv17/ΔCD/ΔUK × Lv17 (green-red × marker-free) virus pair and compared the result with the cross of the Lv17/ΔCD × Lv17/ΔUK (green × red) virus pair. If viruses carrying a single marker gene are crossed, co-infected cells (i.e. being red and green) interfere with the determination of the number of recombinants (also red and green), whereas in reciprocal crosses, a distortion in the quantification of the number of parental viruses will occur. If co-infected cells were to occur in large numbers during titration of the cross, there would be a significant difference between the results of the ‘normal cross’ and the reciprocal cross. However, we measured qRF^3-4^of 20.66% and 17.11% in the two crosses (Figure 5C). No significant difference was revealed in the comparison between crosses of single-deleted parental strains (Lv17/ΔCD and Lv17/ΔUK) and crosses between double-deleted and wild-type parental strains (Lv17 and Lv17/ΔCD/ΔUK) (Mann–Whitney U test, *p* = 0.4363; *n* = 24 and 3, respectively), confirming that the crossing method is reproducible.

**Figure 5.**
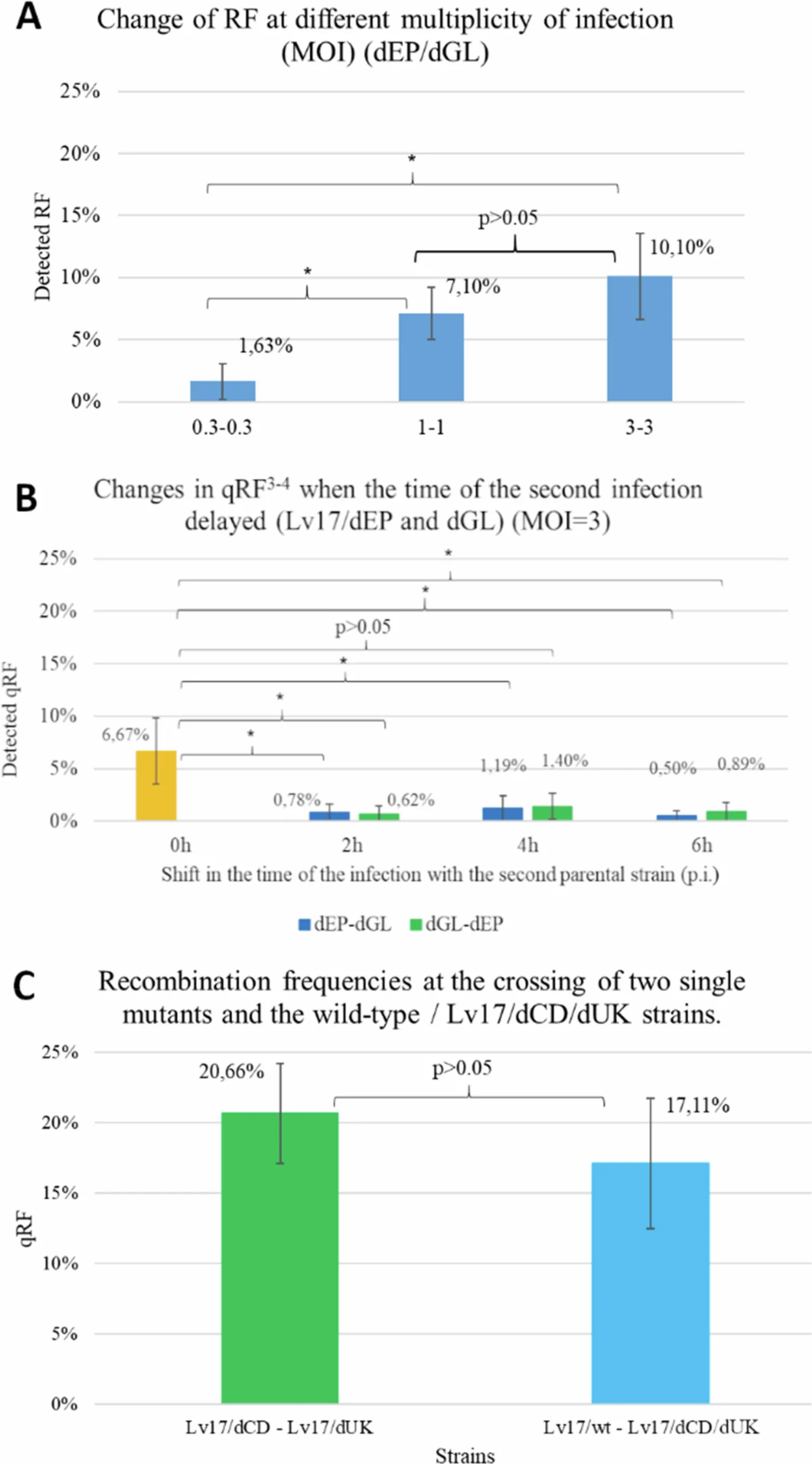
Factors influencing the qRF^3-4^. Each measurement was performed at least in triplicate. Statistical significance was evaluated using two-sample t-tests. *: *P*  ≤ 0.05; **: *P* ≤ 0.01; ***: *P* ≤ 0.001. (A) The qRF^3-4^ decreased significantly if the MOI was lower than 1–1. *P* = 0.0172 between the 3 MOI (*n* = 3) and 0.3 MOI (*n* = 3), and *P* = 0.02023 between 1 MOI (*n* = 3) and 0.3 MOI. (B) The qRF^3-4^ decreased in asynchronous infections. *P* = 0.02781 and 0.02506 after a two-hour delay in infection (Lv17/ΔEP-Lv17/ΔGL and Lv17/ΔGL-Lv17/ΔEP, respectively) compared to synchronous infection (*n* = 4). *P* = 0.03861 and 0.08478 after a 4-hour shift in infection, and *P* = 0.02228 and 0.02963 after a 6-hour delay (*n* = 3 in all cases). (C) The qRF^3-4^ values did not differ significantly in co-infection experiments including either single-deleted parental strains or double-deleted/wild-type parental strains (*p* = 0.4363; *n* = 24 and 3, respectively).

### Study of quasi recombination frequency

After validating the method, we determined the dependence of qRF^3-4^ on the genetic distance between marker genes by crossing seven Lv17 strains with different markers (Figure 6). The value of qRF^3-4^ increased with distance. A saturation curve similar to the Haldane mapping function can be fitted to the measurement points, peaking at approximately 20% qRF^3-4^ at a distance of approximately 160,000 nucleotides (nt). The observed recombination rate saturates at ~20% owing to linkage disequilibrium (LD) (i.e. the loci do not recombine independently), the limited rate of co-infection, and the relatively small number of replication cycles.

**Figure 6.**
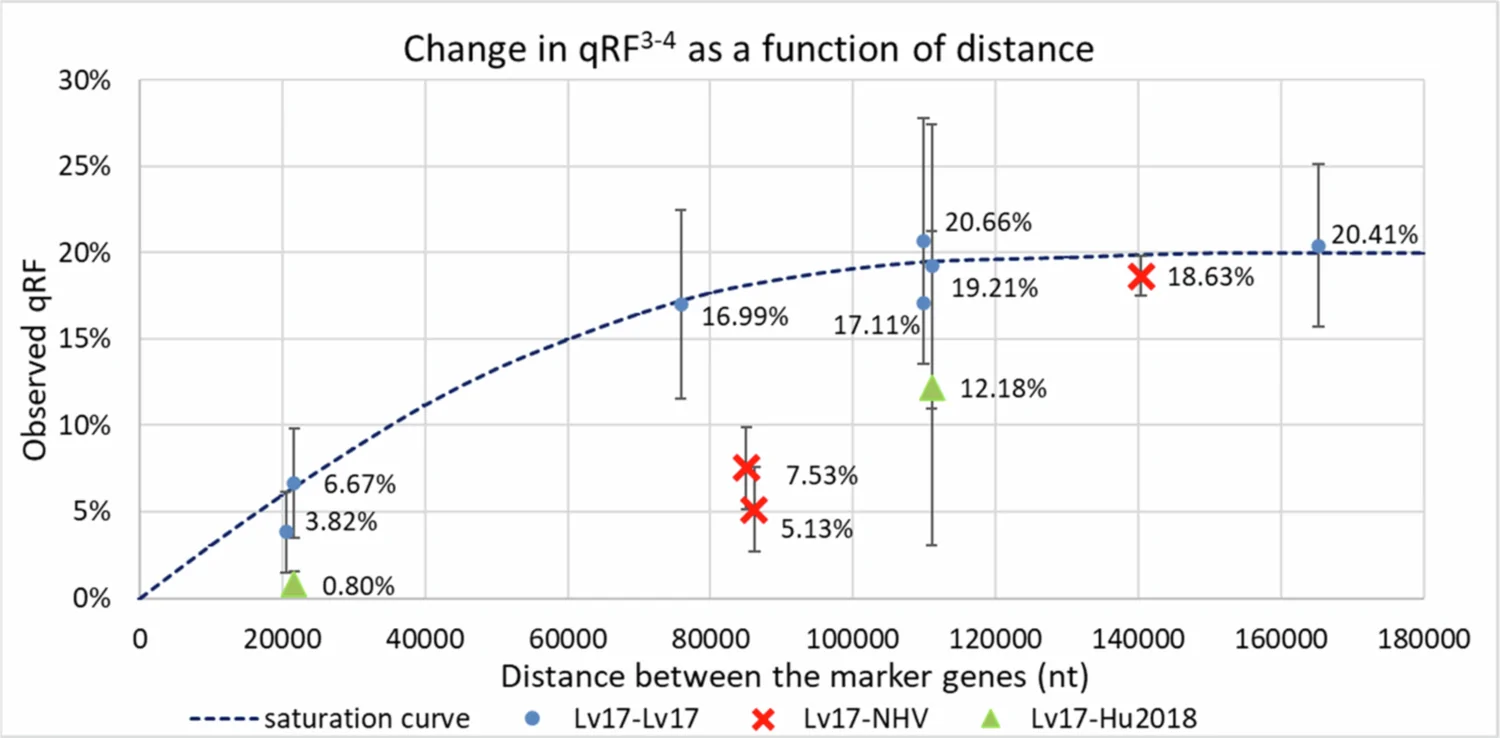
The change in recombination frequency as a function of the distance between marker genes in the two parental strains. The blue dots show the results of the crosses involving the same strain (Lv17), the red crosses represent crosses between different genotypes (genotypes I and II), and the green triangles indicate the crosses with different genotype II strains (Lv17 and Hu2018). The dashed line represents a saturation curve fitted to the data points of the Lv17 crosses (blue dots). Each measurement was performed in triplicate.

Crosses involving different viruses of the same genotype (Lv17 × Hu2018) resulted in lower recombination frequencies at identical distances than those with the same virus background (i.e. Lv17 only) (Figure 6). The qRF^3-4^ values obtained from crosses between biotypes 1 and 2 – which share even lower genetic homology (Lv17 × NHV) – deviated further from the Lv17 curve at a distance of 85 kbp, prior to the saturation point. However, at a larger distance (140 kbp) between marker genes that approached the saturation point (160 kb), the qRF^3-4^ value was only slightly lower than the value expected based on the curve derived from the crosses between homologous virus strains (0.18 vs 0.2).

### Modeling the recombination

We established a simplified mathematical model and wrote a script (Supplementary script 1) to estimate the recombination frequency during ASFV infection in PAMs from Lv17 crosses. The viral population is considered to have two genetic markers, denoted by red (R) and green (G). Recombinant viruses carry both markers (RG), while wild-type viruses (WT) carry none. Recombinants emerge across generations under limited co-infection, where recombination can only occur in cells infected by more than one virus.

At generation (*t* = 0), the viral population consists of:

-
$f_{G\left(0\right)}=0.5$ (green-only viruses)
-
$f_{R\left(0\right)}=0.5$ (red-only viruses)
-
$f_{\mathrm{RG}}(0)=0$ (recombinants)
-
$f_{\mathrm{WT}}(0)=0$ (wild type)

with normalization:

$$f_{G}+f_{R}+f_{\mathrm{RG}}+f_{\mathrm{WT}}=1$$

At each generation, a fraction c∈[0,1] of the viruses enters co-infected cells (i.e. two virions per cell). The remaining fraction (1- c ) infects cells singly. Co-infection is random, meaning that the probability that two viruses of types (*i*) and (*j*) co-infect the same cell is proportional to:

$$P\left(i,j\right)=\;\left\{ \begin{array}{llc} {f_{i}}^{2} & \mathrm{if} & i=j\\ {2f}_{i}f_{j} & \mathrm{if} & i\neq j \end{array}\right.$$

Recombination occurs only in co-infected cells, and it is visible only when the two infecting viruses differ by their markers. Let r∈[0,1] denote the recombination probability per co-infected cell (this is the quantity related to genetic distance, e.g. via the Haldane function).

The outcome might be the following. If both viruses are identical (e.g. (G+G), (R+R)), no detectable recombination occurs. If viruses differ (e.g. (G+R), (WT+RG), etc.), then: with probability r , recombination occurs, and thus, recombinant types are produced. With probability 1−r parental types are preserved.

We assume that each infected cell (whether single or co-infected) produces the same number of progeny virions. Therefore, co-infection does not increase or decrease total output, only redistributes genotype frequencies. After each generation, the genotype frequencies are renormalized.

The recursion equations are the following. Let:

$$f\left(t\right)\;≔\;(f_{G},f_{R},f_{\mathrm{RG}},f_{\mathrm{WT}})$$

denote the frequencies of the 4 possible types at generation *t*. The contribution from non-co-infected fraction is simply:

(1−c)f(t)

(no change in genotype).

From the co-infected fraction (*c*), we sum over all pairings:

$$\sum_{\mathrm{ij}}P\left(i,j\right)O(i,j)$$

where O(i,j) is the offspring distribution vector resulting from infection by types *i* and *j*. For example,

-
$(G\;+\;R):\;O\left(G,R\right)=\left(1-r\right)\left(\frac{1}{2},\frac{1}{2},0,\;0\right)+r\left(0,\;0,\frac{1}{2},\frac{1}{2}\right)$

-
(G+R):O(G,R)=(1, 0, 0, 0) ,

(and similarly for other combinations). Then the full update is:

$$f\left(t+1\right)=\left(1-c\right)f\left(t\right)+\;\sum_{i,j}P\left(i,j\right)O(i,j),$$

followed by normalization. The interpretation of the symbols in the equations is the following:

- (c) controls the rate at which recombination opportunities arise.
- (r) controls the efficiency of recombination per opportunity.
- The model naturally produces:
  ∘ slow buildup of recombinants when (c) is small,
  ∘ distance-dependent recombination via (r(d)),
  ∘ and a nontrivial genotype mixture after a few generations.

The model has the following properties that explain the observed phenomena. The observed recombination as a function of genetic distance reflects the parameter (r(d)). The incomplete saturation (e.g. plateau at ~0.2 instead of 0.5) after a limited number of generations arises from the incomplete co-infection (c < 1), and a limited number (3–4) of replication cycles.

**Figure 7.**
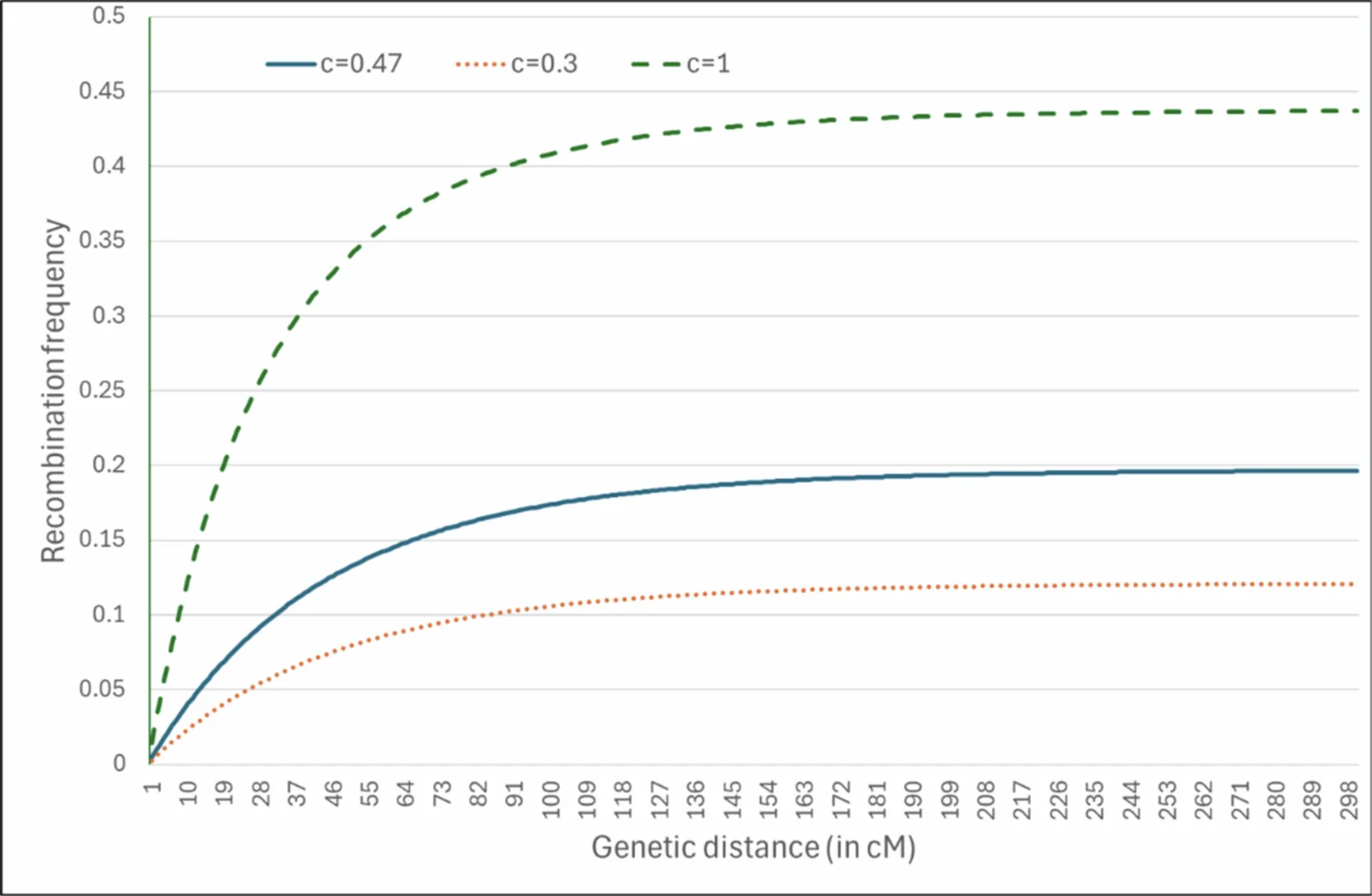
The observed ~0.2 recombination rate after three generations in our co-infection model with co-infection rate c = 0.47. Curves with c = 0.3 and with c = 1 are also plotted for reference. The recombination rates were obtained from the centimorgan (cM) distances according to the Haldane model.

Applying the model and using the observed qRF^3-4^ plateau value of 0.2, the average co-infection rate after three viral cycles is estimated to be 0.47 (Figure 7). This value is a relatively good approximation of the co-infection rates we detected after 2–3 cycles between 40 and 48 h post-infection (~0.35, 0.41; 0.42, 0.32; Figure 3B and D, respectively), confirming the model’s relative reliability.

Based on our results, it can be stated that ASFV recombines extremely efficiently in PAMs under *ex vivo* conditions. Depending on the marker distance and MOI, the proportion of recombinant viruses can exceed 20% among strains of the same origin (100% homology) after three to four virus cycles, and homologous recombination peaks in a manner reminiscent of the Haldane function. Although sequence similarity significantly influences the RF, a high frequency of homologous recombination can still be detected among distant markers, even among strains belonging to different biotypes with decreased homology (~98% sequence similarity between Lv17 and NHV).

## Discussion

The aim of this study was to characterize and provide evidence for the recombination ability of the ASF virus *ex vivo*. Our objective would have been best served by accurately determining the ratio of parental and recombinant viruses in the first offspring generation to calculate the precise recombination frequency. While this is difficult with other viruses, it is nearly impossible in the case of ASFV cultured in PAMs. Several independent obstacles make the accurate determination of the RF in the first virus generation extremely challenging. Even with a high multiplicity of infection (MOI = 10), it is impossible to infect most naïve macrophages simultaneously (Lithgow et al. 2014). Depending on the strain and cell preparation, the proportion of infected macrophages varies from 10% to 60% at 24 h post-infection (hpi). Typically, the number of viruses produced in the first viral cycle (20 hpi) in the infected cell supernatant is too low to distinguish them from the 'contaminating' viruses of the inoculum that did not contribute to the infection (Koltsova et al. 2021).

Furthermore, the proportion of co-infected naïve PAMs remained very low (approximately 0.5% under the applied conditions), even at a high infection rate of 3 MOI (Figure 3). Consequently, a significant proportion of progeny viruses is produced in cells infected by only one parental virus. These viruses then increase the total count of parental viruses in the supernatant, while they or their ancestors did not participate in the production of recombinants. Since the RF is calculated as the ratio of recombinant progeny to total progeny (Wei et al. 2020), the reasons listed above lead to a significant underestimation of the true RF.

In addition to host cell-dependent effects, using different viral strains is another important factor that complicates the accurate determination of the RF. The use of mutant viruses carrying genetic markers is unavoidable for the study of recombination, but this approach introduces an inherent uncertainty related to viral fitness. In most cases, disruption of marker genes is accompanied by alterations in viral fitness (Gladue et al. 2021; Vuono et al. 2021; Petrini et al. 2023b; Ramirez-Medina et al. 2023, p. 240; Tamás et al. 2023), which may influence the recombination frequency and the number of resulting recombinant progeny viruses through effects on viral infectivity and/or replication efficiency. This remains a potential confounding factor even though such fitness effects may be partially mitigated by complementation of the deleted ASFV genes during co-infection, as well as by applying a limited number of replication cycles, such as those employed in our experiments.

### Ex vivo co-infection and recombination

Based on our previously published data (Tamás et al. 2023), we estimate that approximately 20,000 viral genomes can be produced in a single PAM cell during one virus cycle, which corresponds to approximately 14 logarithmic DNA replication cycles (2^14.3^ approximately 20,000). We have limited knowledge about how recombination occurs between genomes during virus replication in a cell, but it is clearly a complex process. It is influenced by numerous biological factors, including the time of infection, virus titer, homology between the parental strains, the fitness of parental strains, and the susceptibility of PAMs towards each of the parental strains.

It is well documented that almost all cells in a macrophage culture become infected in subsequent virus cycles following the initial exposure to the virus (Olasz et al. 2019). Theoretically, at a high MOI infection where more than two viral particles can infect a single cell, the co-infection rate can exceed 50%, while at a random two-particle infection, the co-infection rate is 50%. However, in naïve PAMs, the observed co-infection rate remains significantly lower (<10%, Figure 3B,D) than what would be expected from an interference-free co-infection. Therefore, we can conclude that in the majority of naïve PAMs, only a single viral particle initiates replication. In contrast, later replicative cycles show a significant increase in the proportion of co-infected and/or recombinant virus-infected cells (Figure 3). This shift is probably due to the unique infection dynamics and the morphological variability of ASFV. Among the three distinct forms of ASFV particles (Breese and DeBoer 1966; Salas and Andrés 2013; Gao et al. 2023), mainly the extracellular and intracellular forms of the virus may participate in the initial infection of naïve cells. In subsequent cycles, however, virus-filled apoptotic bodies from disintegrating infected macrophages, macrophage‒macrophage vesicle exchange (Hu et al. 2021), and synaptic-like connections between macrophages (Droesbeke et al. 2025) could also contribute to the observed increase in infection rates. If any or all of these processes drive the increase in co-infection, they should be much more efficient at inducing simultaneous particle uptake and subsequent recombination than the first two routes. Alternatively, macrophages in an already infected culture may become more susceptible to simultaneous multiple infections during subsequent cycles owing to cytokine and chemokine release. Under *ex vivo* conditions, significant accumulation of the chemokines CXCL10, CCL2, and IL-8 can be measured in PAMs in response to ASFV (Fishbourne et al., 2013). These factors may serve as a chemotactic gradient, while the paracrine effect of the released cytokines may increase the susceptibility of adjacent macrophages to simultaneous infections. Determining the relative impact of these biological processes on co-infection and recombination remains a significant challenge.

It is also somewhat surprising that the synchrony of the initial infection plays such a large role in the development of the qRF^3-4^ value (Figure 5B), as merely 2 h of asynchronization reduced the proportion of recombinant offspring by 90%. This sharp decline likely stems from the synergistic effect of several interdependent biological processes. Relatively high-titer (MOI = 3) infections may swiftly saturate the receptors involved in ASFV infection with virus particles, facilitating immediate virus uptake. Given that, in tissue culture, ASFV virions internalize into late endosomes within 30–40 min post-infection, and the first proteins appear within 2–4 h after infection (Hooper et al. 2024), the first virus has a significant temporal advantage, even if the viruses administered 2 h later were to enter and replicate unhindered in PAMs. However, this is highly unlikely to happen. As with most viruses, cells in the eclipse phase cannot be infected by identical or closely related viruses (Folimonova 2012; Bergua et al. 2014). This phenomenon is known as superinfection exclusion. For this reason, with asynchronous infection, more cells can be infected in the first virus cycle with the first virus than with the second virus added 2 h later. The incidence of co-infection is highest when the two virus strains are present in a 50–50 ratio. Any deviation from this ratio leads to a decrease in the number of co-infections and related recombination events, which can accumulate over 3–4 cycles and can lead to a significant reduction of recombinants observed in our study.

Recombination, viral replication, and DNA repair are interconnected processes in which viruses use host- or virus-encoded machinery to repair damaged genomes, restart stalled replication forks, and promote genetic diversification (Sakakibara et al. 2013; Pérez-Losada et al. 2015). The exact molecular mechanism of ASFV homologous recombination is currently unknown. However, as previously discussed (Petrini et al. 2023a, p. 17), it is likely that single-strand invasion is the predominant mechanism mediating homologous recombination, as in other DNA viruses and DNA-based genomes. A reduction in sequence homology markedly decreases the recombination frequency because homologous recombination requires sufficient uninterrupted sequence identity to stabilize and process strand invasion intermediates. Consequently, sequence divergence or shorter homologous regions substantially reduce recombination efficiency. The overall nucleotide identity between the Lv17 and NHV strains is approximately 98%. Our results suggest that this 2% divergence significantly reduces homologous recombination, even over relatively long genomic distances. Instead of the expected 17–18% qRF^3-4^ value observed between fully homologous genomes, we measured only 5–8% qRF^3-4^ between genotype I and II viruses (NHV and Lv17, respectively) at a distance of ~85 kb. As the distance increases and the number of fully homologous regions expands, this effect diminishes. At 140 kb, qRF^3-4^ approached the value measured between homologous strains (0.18 vs. 0.2). Further investigation is needed to determine the extent to which increasing genetic divergence reduces homologous recombination between ASFV strains, and whether recombinant progeny can be generated between highly divergent isolates sharing only 75.4% genome similarity (Bao et al. 2022), such as Kenya 1950 (genotype X, biotype 4) and ASFV/NH/P68 (genotype I, biotype 1) (Dinhobl et al. 2023).

Co-infection experiments allowed us to compare the recombination rates between a *GL*-deleted Lv17 strain and two others: one with a deleted *EP153R* gene and another where both the neighboring *EP153R* and *EP402R* had been knocked out simultaneously (Figure 2). In the co-infection experiment with the double-gene knockout virus, the distance between the marker genes decreased from 21,000 nt to 20,000 nt (less than 5%) compared to the situation in which the single-gene deleted virus was included, while the recombination frequency was reduced from 6.67% to 3.82% (more than 40%) (Figure 5). Despite the lack of a statistically significant difference (*P* = 0.2507), these results align with earlier *in silico* findings suggesting that the *EP402R* gene contains a recombination hotspot and that its deletion might reduce the recombinogenic potential of the virus in this region (Zhu et al. 2019; Nefedeva et al. 2020).

### Modeling ASFV recombination

We created a mathematical model that fits our data and allows us to estimate the effect of several key factors (genetic distance, co-infection rate, and number of generations) on ASFV recombination.

Our model has a very interesting aspect. The maximum homologous recombination frequency that can be observed between two genetic loci during sexual reproduction is 50%. In our model, even if the co-infection rate were 1, and the recombination rate reached its maximum value (*r* = 0.5), the RF would be only 0.25 instead of 0.5 in one generation. Since virions are taken at random during the co-infection process, there is a 50% probability of green and red virions entering the cells together. Meanwhile, the probability of two green or two red virions entering the cells is 0.25–0.25. Since recombination is detected only when different virions get into the cell in the co-infection procedure, the rate of recombinants after one generation is 0.25 (the probability of a cell being co-infected by a red and a green virion (0.5) multiplied by the recombination frequency in that cell (0.5)).

Given the limited sample size, unquantified fitness differences among strains, and high experimental variability, only a coarse estimation of the relationship between physical distance (bp) and genetic distance (cM) can be achieved. In Figure 6, the observed plateau of the recombination curve (~20.4% qRF^3-4^) occurs at a marker distance slightly exceeding 165,000 nucleotides. According to our mathematical model (Figure 7), this plateau corresponds to an estimated genetic distance on the order of 100–300 cM after three to four replication cycles. Dividing the corresponding physical distance (~165 kbp) by this estimated genetic distance yields an approximate conversion of 550–1650 bp per cM. We emphasize that the 100–300 cM estimate was obtained by qualitative interpretation of the model and the observed saturation behavior, rather than by formal parameter fitting. Therefore, these values should be regarded as approximate and indicative rather than precise quantitative estimates.

Consequently, we estimate that offspring virions originating from a single cell undergo, on average, 1.15–3.45 genome-wide recombination events. We stress that this estimate is based on a qualitative, ‘by eye’ interpretation rather than a formal fit and should be regarded as indicative rather than precise quantitative data.

### Implications for in vivo recombination

Although these experiments were conducted *ex vivo*, it is important to consider how the observed processes manifest *in vivo* and what their potential implications might be, especially regarding disease prevention. Even without accounting for corrective factors, the qRF^3-4^ value of 0.2 measured between homologous strains and the nearly identical qRF^3-4^ value of 0.18 obtained from crosses between two different biotypes should be alarming (Figure 6). These values indicate that after only 3‒4 viral replication cycles, 18%–20% of all progeny ASFVs are recombinant within a population of just ~10^5^ cells. We know that in a 20–40 kg pig, the broncho-alveolar lavage contains approximately 8 × 10^8^ PAMs, and 1  cm^3^ of porcine lymph node can contain up to 2 × 10^8^ macrophages (Carrascosa et al. 1982). While the total macrophage count for swine has not yet been established, it is estimated to be 2 × 10^11^ for the similar human immune system. These numbers and the packed nature of immune cells in the lymph nodes indicate a very high number of macrophage–macrophage interactions, which can exceed those achieved in the *in vitro* environment by orders of magnitude. Therefore, significantly more infectious cycles can occur within a host than in our *ex vivo* experiments, which may lead to a much larger proportion of recombinant viruses because of LD breakdown. LD is the distance-dependent association of linked alleles at different loci within a population, and it decays with each generation in a freely combining population due to genetic recombination (Hui and Burt 2020).

A high *in vivo* recombination rate among different ASFV strains can lead to the emergence of recombinants with increased fitness and/or virulence. This complicates the epidemiological situation and hinders disease control efforts. Furthermore, *in vivo* experiments provided additional evidence that the threat posed by recombination in the field is real and should not be underestimated. Notably, co-infecting pigs with two attenuated strains (OUR T88/3 and AQSΔB119L) belonging to different genotypes (type I and type II, respectively) led to symptoms characteristic of virulent ASFV infections (Kitamura et al. 2025). The genome sequences of twelve ASFV isolates obtained from four experimentally infected pigs revealed significant genetic diversity and various chimeric patterns, demonstrating that high recombination frequencies can occur not only *in vitro* but also *in vivo* during co-infection between relatively distant ASFV genotypes.

Outside of Africa, the risk of co-infection with different genotypes was limited until recently. However, three virulent genotype I-II recombinant ASFVs were isolated in the northeastern region of China in 2021–2022, shortly after the first genotype I strains were isolated in the country (Zhao et al. 2023). Sequence analysis of the isolates revealed a mosaic genome consisting of 10 large genotype I and 10 genotype II fragments. Approximately 80.7 kbp of the recombinant genome showed 99.36–100% identity to the homologous genome fragments of the genotype I SD/DY-I/21 strain, while 104.7 kbp was 99.95–100% identical to the homologous genome fragments of the genotype II HLJ/18 strain. The emergence of this chimera may have been facilitated by changes in its immunological properties relative to those of at least one of its parental strains. The recombinant virus JS/LG/21 can evade immunity induced by genotype II live-attenuated ASFV vaccines, spreads rapidly, and has already been detected in Russia and Vietnam (Igolkin et al. 2024; Le et al. 2024). This strongly suggests that co-infection-facilitated recombination and the emergence of recombinants with altered biological phenotypes are not just a theoretical risk but a real danger when different ASFV strains circulate in the same geographical area. Vaccination with MLVs will inevitably create scenarios where a new strain is introduced into or around an already infected area. Therefore, the risks of co-infection and recombination must be rigorously evaluated by both vaccine developers and regulatory authorities to implement necessary mitigation steps.

### Reducing the risk of in vivo recombination

Obviously, the best way to limit recombination is to reduce the likelihood of *in vivo* co-infection. Consequently, the use of MLVs for disease prevention is not the optimal approach. Unfortunately, they are currently the only available option for vaccination. Considering this, immunizing an animal with an MLV vaccine that results in low viral loads, rapid vaccine virus clearance, and minimal vaccine virus shedding poses a lower risk than a vaccine lacking these features. MLVs can recombine in the field with circulating strains and with each other, which increases the genetic diversity of ASFV (Figure 8). Most MLV vaccine candidates carry multiple deletions distributed across the viral genome. The combined effects of these deletions are required to achieve sufficient safety and efficacy. Individual mutations or deletions are generally insufficient for a safe and effective vaccine. However, if recombination results in the separation of attenuating mutations, recombinant viruses will be created with only a subset of the original gene deletions. These viruses may lose attenuation or fail to meet safety requirements, giving rise to novel pathogenic strains (Figure 8B). Therefore, taking recombination into account, a live vaccine is safer if its genetic modification is limited to a single region (Figure 8A) rather than to multiple regions (Figure 8B).

Moreover, recombination can lead to the loss of a DIVA marker from a vaccine strain. It can also facilitate the integration of the DIVA marker into wild-type viral genomes and poses a problem for performing reliable DIVA diagnostics.

Of course, the safety of MLVs is determined by several factors, and recombination is only one of them; others are equally important. For example, the ASFV-G-ΔI177L mutant has only a single gene deletion, but it is genetically unstable and considered an unsafe ASF vaccine (van den Born et al. 2025; Friedrichs et al. 2026).

**Figure 8.**
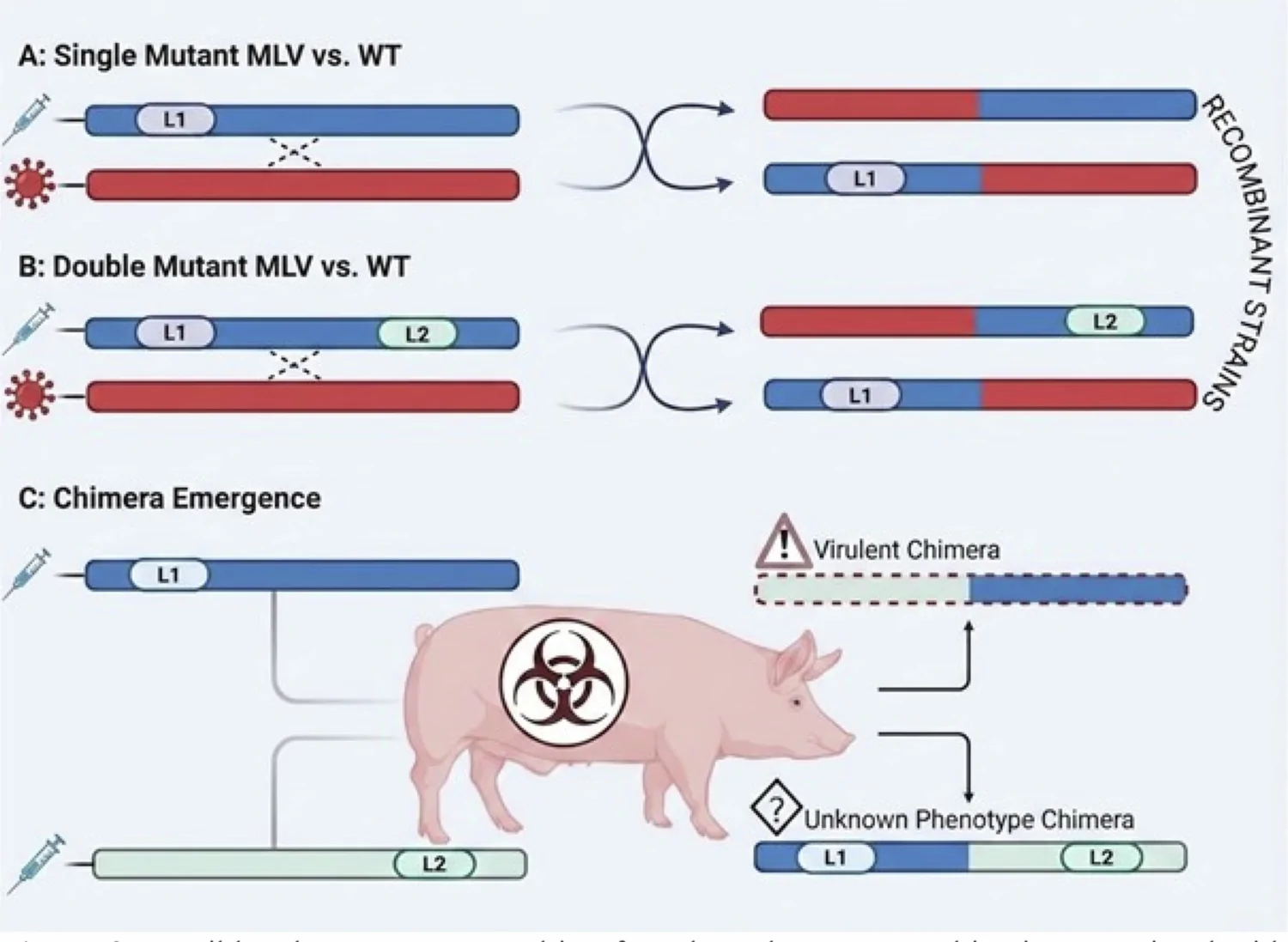
Possible adverse events resulting from homologous recombination associated with vaccination. L1 and L2 represent modified regions contributing to vaccine safety. (A) Recombination of the MLV with a circulating field strain. (B) Loss of attenuation of the double-gene-deleted MLV, potentially resulting in the emergence of novel pathogenic strains. (C) Following vaccination with two MLVs, the emergence of virulent and double-gene-deleted strains with unknown phenotypes.

In the event of ASFV outbreaks, a control area and a vaccination ring area should be established so that vaccinated animals have a minimal chance of encountering the wild-type virus during the vaccine clearance period, thereby excluding or minimizing the risk of co-infection. Using more than one MLV with modifications at different genome loci within a single geographical area could be a costly mistake and a high-risk endeavor. This is because accidental co-infection of the vaccines can lead to the emergence of virulent wild-type virus or pathogenic chimeras, depending on the number of modified loci and the genotype of the vaccine strains (Figure 8C). Consequently, we propose the authorization of the use of a single MLV rather than multiple MLVs for prevention within ASFV-affected regions. Since 2022, three MLVs (ASFV-G-∆I177L, ASFV-G-∆MGF, and ASFV-G-∆I177L/∆LVR) have received marketing authorization in Vietnam and the Philippines (Borca et al. 2020; Nguyen et al. 2025; Friedrichs et al. 2026). Although there have been reports of safety issues (Nguyen et al. 2025; van den Born et al. 2025), the emergence of vaccine-derived chimeras or recombinants has not been reported. In any case, authorities in other countries should closely monitor developments in the Vietnamese epidemic and draw appropriate conclusions.

## Data Availability

The nucleotide sequence data generated in this study have been deposited in the National Center for Biotechnology Information (NCBI) database. The raw sequence data of the project are deposited in the NCBI BioProject database (ID: PRJNA1503432). The Python analysis script has been deposited in the ARP Data Repository and can be accessed via the following link: https://hdl.handle.net/21.15109/ARP/NPOFMG.

## References

[cit0001] Afonso CL , Carrillo C , Zsak L , Rock DL , Borca MV . 1998. African swine fever virus NL gene is not required for virus virulence. J Gen Virol. 79:2543–2547. 10.1099/0022-1317-79-10-2543 9780062

[cit0002] Alejo A , Matamoros T , Guerra M , Andrés G . 2018. A proteomic Atlas of the African swine fever virus particle. J Virol. 92:e01293-18. 10.1128/JVI.01293-18 PMC6232493 30185597

[cit0003] Arias M et al. 2017. Approaches and perspectives for development of African swine fever virus vaccines. Vaccines. 5:35. 10.3390/vaccines5040035 PMC5748602 28991171

[cit0004] Bao J et al. 2022. Genome-wide diversity analysis of African swine fever virus based on a curated dataset. Anim Open Access J MDPI. 12:2446. 10.3390/ani12182446 PMC9495133 36139306

[cit0005] Barasona JA et al. 2021. Safety of African swine fever vaccine candidate Lv17/WB/Rie1 in wild boar: overdose and repeated doses. Front Immunol. 12:761753. 10.3389/fimmu.2021.761753 PMC8669561 34917082

[cit0006] Bergua M et al. 2014. A viral protein mediates superinfection exclusion at the whole-organism level but is not required for exclusion at the cellular level. J Virol. 88:11327–11338. 10.1128/JVI.01612-14 PMC4178825 25031351

[cit0007] Borca MV et al. 2020. Development of a highly effective African swine fever virus vaccine by deletion of the I177L gene results in sterile immunity against the current epidemic eurasia strain. J Virol. 94:e02017-19. 10.1128/JVI.02017-19 PMC7081903 31969432

[cit0008] Borca MV , Holinka LG , Berggren KA , Gladue DP . 2018. CRISPR-Cas9, a tool to efficiently increase the development of recombinant African swine fever viruses. Sci Rep. 8:3154. 10.1038/s41598-018-21575-8 PMC5816594 29453406

[cit0009] van den Born E et al. 2025. African swine fever virus vaccine strain Asfv-G-∆I177l reverts to virulence and negatively affects reproductive performance. NPJ Vaccines. 10:46. 10.1038/s41541-025-01099-9 PMC11885574 40050309

[cit0010] Breese SS , DeBoer CJ . 1966. Electron microscope observations of African swine fever virus in tissue culture cells. Virology. 28:420–428. 10.1016/0042-6822(66)90054-7 18611474

[cit0011] Cadenas-Fernández E et al. 2020. Adenovirus-vectored African swine fever virus antigens cocktail is not protective against virulent Arm07 isolate in eurasian wild boar. Pathogens. 9:171. 10.3390/pathogens9030171 PMC7157622 32121082

[cit0012] Carrascosa AL , Santarén JF , Viñuela E . 1982. Production and titration of African swine fever virus in porcine alveolar macrophages. J Virol Methods. 3:303–310. 10.1016/0166-0934(82)90034-9 7085838

[cit0013] Chapman DAG , Tcherepanov V , Upton C , Dixon LK . 2008. Comparison of the genome sequences of non-pathogenic and pathogenic African swine fever virus isolates. J Gen Virol. 89:397–408. 10.1099/vir.0.83343-0 18198370

[cit0014] Dinhobl M et al. 2023. Reclassification of ASFV into 7 biotypes using unsupervised machine learning. Viruses. 16:67. 10.3390/v16010067 PMC10819123 38257767

[cit0015] Dong L et al. 2026. Recombinant vaccinia vectored ASFV vaccine enhances swine survival against genotype II challenge. NPJ Vaccines. 11:58. 10.1038/s41541-026-01377-0 PMC12932654 41577679

[cit0016] Droesbeke B , Balmelle N , Nauwynck HJ , Favoreel H , Tignon M . 2025. African swine fever virus infection of porcine peripheral blood monocyte-derived macrophages induces the formation of tunneling nanotube-connected large vesicle-like cell segments: a potential mechanism for intercellular ASFV trafficking. Vet Res. 56:148. 10.1186/s13567-025-01582-0 PMC12247315 40640946

[cit0017] Fan J , Yu H , Miao F , Ke J , Hu R . 2024. Attenuated African swine fever viruses and the live vaccine candidates: a comprehensive review. Microbiol Spectr. 12:e0319923. 10.1128/spectrum.03199-23 PMC11537121 39377589

[cit0018] Folimonova SY . 2012. Superinfection exclusion is an active virus-controlled function that requires a specific viral protein. J Virol. 86:5554–5561. 10.1128/JVI.00310-12 PMC3347309 22398285

[cit0019] Franzoni G et al. 2023. Evaluation of haematological and immunological parameters of the ASFV Lv17/WB/Rie1 strain and its derived mutant Lv17/WB/Rie1/d110-11L against ASFV challenge infection in domestic pigs. Vaccines. 11:1277. 10.3390/vaccines11071277 PMC10383595 37515092

[cit0020] Friedrichs V . 2026. Implications for safety: ASFV-G-δI177L vaccine compromises health and semen quality inadult breeding boars. Front Microbiol. 17:1823118. 10.3389/fmicb.2026.1823118 PMC13168198 42137814

[cit0021] Gallardo C et al. 2019. Attenuated and non-haemadsorbing (non-HAD) genotype II African swine fever virus (ASFV) isolated in Europe, Latvia 2017. Transbound Emerg Dis. 66:1399–1404. 10.1111/tbed.13132 30667598

[cit0022] Gallardo C et al. 2024. Double deletion of EP402R and EP153R in the attenuated Lv17/WB/Rie1 African swine fever virus (ASFV) enhances safety, provides DIVA compatibility, and confers complete protection against a genotype II virulent strain. Vaccines. 12:1406. 10.3390/vaccines12121406 PMC11680264 39772067

[cit0023] Gao P et al. 2023. Riding apoptotic bodies for cell-cell transmission by African swine fever virus. Proc Natl Acad Sci U S A. 120:e2309506120. 10.1073/pnas.2309506120 PMC10691326 37983498

[cit0024] Gershon PD , Paul Kitching R , Hammond JM , Black DN . 1989. Poxvirus Genetic Recombination during Natural Virus Transmission. Journal of General Virology. 70:485–489. 10.1099/0022-1317-70-2-485 2543750

[cit0025] Gladue DP et al. 2021. Deletion of the A137R gene from the pandemic strain of African swine fever virus attenuates the strain and offers protection against the virulent pandemic virus. J Virol. 95:e0113921. 10.1128/JVI.01139-21 PMC8513468 34406865

[cit0026] Grishagin IV . 2015. Automatic cell counting with ImageJ. Anal Biochem. 473:63–65. 10.1016/j.ab.2014.12.007 25542972

[cit0027] Hooper GL , Netherton CL , Wright E . 2024. Cell entry mechanisms of African swine fever virus. Virology. 600:110277. 10.1016/j.virol.2024.110277 39488059

[cit0028] Hu Q et al. 2021. Extracellular vesicle activities regulating macrophage- and tissue-mediated injury and repair responses. Acta Pharm Sin B. 11:1493–1512. 10.1016/j.apsb.2020.12.014 PMC8245807 34221864

[cit0029] Hui T-YJ , Burt A . 2020. Estimating linkage disequilibrium from genotypes under hardy-weinberg equilibrium. BMC Genet. 21:21. 10.1186/s12863-020-0818-9 PMC7045472 32102657

[cit0030] Hunter JD . 2007. Matplotlib: a 2D graphics environment. Comput Sci Eng. 9:90–95. 10.1109/MCSE.2007.55

[cit0031] Igolkin A et al. 2024. Detection of the first recombinant African swine fever virus (genotypes I and II) in domestic pigs in russia. Mol Biol Rep. 51:1011. 10.1007/s11033-024-09961-0 39320407

[cit0032] Kitamura T , Masujin K , Ikezawa M , Ambagala A , Kokuho T . 2025. Generation of chimeric African swine fever viruses through in vitro and in vivo intergenotypic gene complementation. Vaccines. 13:462. 10.3390/vaccines13050462 PMC12115777 40432074

[cit0033] Koltsova G et al. 2021. Growth kinetics and protective efficacy of attenuated ASFV strain Congo with deletion of the EP402 gene. Viruses. 13:1259. 10.3390/v13071259 PMC8309992 34203302

[cit0034] Lacasta A et al. 2014. Expression library immunization can confer protection against lethal challenge with African swine fever virus. J Virol. 88:13322–13332. 10.1128/JVI.01893-14 PMC4249112 25210179

[cit0035] Le VP et al. 2024. Detection of recombinant African swine fever virus strains of p72 genotypes I and II in domestic pigs, Vietnam, 2023. Emerg Infect Dis. 30:991–994. 10.3201/eid3005.231775 PMC11060461 38666642

[cit0036] Li Y et al. 2026. The deletion of the EP402R and MGF505/360 genes attenuates a genotype I/II recombinant ASFV but fails to confer complete protection against homologous or genotype II challenge in pigs. Emerg Microbes Infect. 15:2608396. 10.1080/22221751.2025.2608396 PMC12798666 41427789

[cit0037] Lithgow P , Takamatsu H , Werling D , Dixon L , Chapman D . 2014. Correlation of cell surface marker expression with African swine fever virus infection. Vet Microbiol. 168:413–419. 10.1016/j.vetmic.2013.12.001 PMC3969584 24398227

[cit0038] Lokhandwala S et al. 2016. Induction of robust immune responses in swine by using a cocktail of adenovirus-vectored African swine fever virus antigens. Clin Vaccine Immunol. 23:888–900. 10.1128/CVI.00395-16 PMC5098023 27628166

[cit0039] Ma B et al. 2025. Deletion of the African swine fever virus E120R gene completely attenuates its virulence by enhancing host innate immunity and impairing virus release. Emerg Microbes Infect. 14:2555722. 10.1080/22221751.2025.2555722 PMC12451965 40899401

[cit0040] Michaud V , Randriamparany T , Albina E . 2013. Comprehensive phylogenetic reconstructions of African swine fever virus: proposal for a new classification and molecular dating of the virus. PLoS One. 8:e69662. 10.1371/journal.pone.0069662 PMC3723844 23936068

[cit0041] Nefedeva M , Titov I , Tsybanov S , Malogolovkin A . 2020. Recombination shapes African swine fever virus serotype-specific locus evolution. Sci Rep. 10:18474. 10.1038/s41598-020-75377-y PMC7794389 33116230

[cit0042] Neilan JG et al. 2004. Neutralizing antibodies to African swine fever virus proteins p30, p54, and p72 are not sufficient for antibody-mediated protection. Virology. 319:337–342. 10.1016/j.virol.2003.11.011 14980493

[cit0043] Nguyen TC et al. 2025. An African swine fever vaccine-like variant with multiple gene deletions caused reproductive failure in a Vietnamese breeding herd. Sci Rep. 15:14919. 10.1038/s41598-025-95641-3 PMC12037777 40295549

[cit0044] O’Donnell V et al. 2015. African swine fever virus Georgia isolate harboring deletions of MGF360 and MGF505 genes is attenuated in swine and confers protection against challenge with virulent parental virus. J Virol. 89:6048–6056. 10.1128/JVI.00554-15 PMC4442422 25810553

[cit0045] O’Donnell V et al. 2017. Simultaneous deletion of the *9gl* and *uk* genes from the African swine fever virus Georgia 2007 isolate offers increased safety and protection against homologous challenge. J Virol. 91:e01760-16. 10.1128/JVI.01760-16 PMC5165186 27795430

[cit0046] Olasz F et al. 2019. A simple method for sample preparation to facilitate efficient whole-genome sequencing of African swine fever virus. Viruses. 11:1129. 10.3390/v11121129 PMC6950082 31817647

[cit0047] Olasz F et al. 2020. Short and long-read sequencing survey of the dynamic transcriptomes of African swine fever virus and the host cells. Front Genet. 11:758. 10.3389/fgene.2020.00758 PMC7399366 32849785

[cit0048] Pérez-Losada M , Arenas M , Galán JC , Palero F , González-Candelas F . 2015. Recombination in viruses: mechanisms, methods of study, and evolutionary consequences. Infect Genet Evol J Mol Epidemiol Evol Genet Infect Dis. 30:296–307. 10.1016/j.meegid.2014.12.022 PMC7106159 25541518

[cit0049] Petrini S et al. 2023a. The production of recombinant African swine fever virus Lv17/WB/Rie1 strains and their in vitro and in vivo characterizations. Vaccines. 11:1860. 10.3390/vaccines11121860 PMC10748256 38140263

[cit0050] Petrini S et al. 2023b. The production of recombinant African swine fever virus Lv17/WB/Rie1 strains and their in vitro and in vivo characterizations. Vaccines. 11:1860. 10.3390/vaccines11121860 PMC10748256 38140263

[cit0051] Python Software Foundation. 2016. Python (Version 3.x) [Computer software]. https://www.python.org

[cit0052] Qin L , Evans DH . 2014. Genome Scale Patterns of Recombination between Coinfecting Vaccinia Viruses. Journal of Virology. 88:5277–5286. 10.1128/jvi.00022-14 PMC4019122 24574414

[cit0053] Ramirez-Medina E et al. 2023. Deletion of the H240R gene in African swine fever virus partially reduces virus virulence in swine. Viruses. 15:1477. 10.3390/v15071477 PMC10384018 37515164

[cit0054] Ramirez-Medina E et al. 2024. Recombinant vaccine strain ASFV-G-δ9GL/δUK produced in the IPKM cell line is genetically stable and efficacious in inducing protection in pigs challenged with the virulent African swine fever virus field isolate Georgia 2010. Pathogens. 13:319. 10.3390/pathogens13040319 PMC11055038 38668274

[cit0055] Rowlands RJ et al. 2008. African swine fever virus isolate, Georgia, 2007. Emerg Infect Dis. 14:1870–1874. 10.3201/eid1412.080591 PMC2634662 19046509

[cit0056] Sakakibara N , Chen D , McBride AA . 2013. Papillomaviruses use recombination-dependent replication to vegetatively amplify their genomes in differentiated cells. PLoS Pathog. 9:e1003321. 10.1371/journal.ppat.1003321 PMC3701714 23853576

[cit0057] Salas ML Andrés G . 2013. African swine fever virus morphogenesis. Virus Res. 173:29–41. 10.1016/j.virusres.2012.09.016 23059353

[cit0058] Sanford B et al. 2016. Deletion of the thymidine kinase gene induces complete attenuation of the Georgia isolate of African swine fever virus. Virus Res. 213:165–171. 10.1016/j.virusres.2015.12.002 26656424

[cit0059] Szeredi L et al. 2020. Detection of African swine fever virus in cell culture and wild boar tissues using a commercially available monoclonal antibody. J Virol Methods. 282:113886. 10.1016/j.jviromet.2020.113886 32450150

[cit0060] Tamás V et al. 2023. Involvement of the MGF 110-11L gene in the African swine fever replication and virulence. Vaccines. 11:846. 10.3390/vaccines11040846 PMC10145817 37112759

[cit0061] Tran XH et al. 2025. African swine fever vaccine ASFV-G-δI177L induces solid protection in four-week-old piglets. Sci Rep. 16:1794. 10.1038/s41598-025-31405-3 PMC12804788 41436821

[cit0062] Umene K . 1999. Mechanism and application of genetic recombination in herpesviruses. Rev Med Virol. 9(3):171–82. 10.1002/(sici)1099-1654(199907/09)9:3<171::aid-rmv243>3.0.co;2-a 10479778

[cit0063] Vuono EA et al. 2021. Evaluation of the function of the ASFV KP177R gene, encoding for structural protein p22, in the process of virus replication and in swine virulence. Viruses. 13:986. 10.3390/v13060986 PMC8227490 34073222

[cit0064] Wei KH-C , Mantha A , Bachtrog D . 2020. The theory and applications of measuring broad-range and chromosome-wide recombination rate from allele frequency decay around a selected locus. Mol Biol Evol. 37:3654–3671. 10.1093/molbev/msaa171 PMC7743735 32658965

[cit0065] Zajac MD et al. 2023. Immunization of pigs with replication-incompetent adenovirus-vectored African swine fever virus multi-antigens induced humoral immune responses but no protection following contact challenge. Front Vet Sci. 10:1208275. 10.3389/fvets.2023.1208275 PMC10316028 37404778

[cit0066] Zhao D et al. 2023. Highly lethal genotype I and II recombinant African swine fever viruses detected in pigs. Nat Commun. 14:3096. 10.1038/s41467-023-38868-w PMC10226439 37248233

[cit0067] Zhu Z et al. 2019. Homologous recombination shapes the genetic diversity of African swine fever viruses. Vet Microbiol. 236:108380. 10.1016/j.vetmic.2019.08.003 PMC7172151 31500735

